# Hair cortisol as outcome parameter for psychological and neuropsychiatric interventions—a literature review

**DOI:** 10.3389/fpsyt.2023.1227153

**Published:** 2023-10-10

**Authors:** Tim Botschek, Vincent Hußlein, Eva M. J. Peters, Burkhard Brosig

**Affiliations:** ^1^Family Psychosomatics, Department of Pediatrics and Neonatology, Justus-Liebig University Giessen, Gießen, Germany; ^2^Psychoneuroimmunology Laboratory, Department of Psychosomatic Medicine and Psychotherapy, Justus-Liebig University Giessen, Gießen, Germany; ^3^Psychosomatics and Psychotherapy, Charité Center 12 Internal Medicine and Dermatology, Charité—Universitätsmedizin Berlin, Berlin, Germany

**Keywords:** hair cortisol, hair cortisol concentration (HCC), psychotherapy research, psychological interventions, outcome, psycho-neuroendocrinology, structured review

## Abstract

**Background:**

Studies measuring hair cortisol concentration (HCC) have been increasingly conducted to document stress-related, endocrine changes aggregated over time. Previous studies have shown that HCC reflects abnormalities in the hypothalamic–pituitary-adrenocortical axis (HPA axis) in the context of somatic diseases, such as Cushing’s syndrome. HCC variations also reveal a corresponding alteration in HPA-axis-function in mental disorders, highlighting its potential role as a biomarker for interventions targeting mental health problems.

**Aims:**

The aim of this study was to investigate the role of HCC in various psychological and neuropsychiatric interventions and to explore the extent to which HCC can serve as a predictive or outcome parameter in such interventions by conducting a PRISMA-compliant review of the literature.

**Methods:**

From May to July 2022, the databases *Web of Science, Google Scholar, PsychINFO*, and *ResearchGate* were systematically searched using different combinations of relevant keywords. Studies of different types that examined HCC in the context of a wide range of psychological and neuropsychiatric interventions were included. Studies in languages other than English or German and animal studies were excluded. The MMAT tool was used, to assesses the Risk of bias.

**Results:**

The initial search identified 334 studies. After applying the inclusion and exclusion criteria, 14 publications with a total number of 1,916 participants were identified. An association between HCC and PTSD, depressive disorders, and ongoing social and family stress can be documented. The effect of relaxation techniques, mental training, CBT, or PTSD therapy on HCC has been studied with equivocal results. Some studies found decreased HCC after treatment, while others did not show a clear effect. Baseline HCC appears to be of particular importance. In some studies, higher baseline HCC was associated with increased treatment response, providing a predictive value for HCC.

**Discussion:**

HCC is increasingly being used as a biomarker for the mapping of psychological and neuropsychiatric interventions. However, due to the wide range of study populations and interventions, results are still heterogeneous. Nevertheless, HCC seems to be an encouraging biological parameter to describe the trajectory of different interventions aimed at improving mental health.

## Highlights

- HCC is increasingly used as trajectory or predictive parameter in psychological interventions.
- The results do not provide a consistent picture to this point.
- However, HCC appears to be a promising biomarker to illustrate how biological processes can be modulated by psychological and neuropsychiatric interventions.
- The methodological gold standard (large, randomized controlled studies with a standardized and comparable study design) for psychotherapy studies with determination of HCC should be implemented more consistently in the future.

## Introduction

Stress has long been recognized as a risk factor for mental and physical health. The hypothalamic–pituitary–adrenal axis (HPA-axis) plays a critical role in the endocrine regulation of stress. Its activation is required for physical adaptation to any stressor and leads to the release of the hormone cortisol (1). To date, blood, saliva, or urine samples have been primarily used to measure cortisol levels. Although these methods are well established and validated, they can only determine current cortisol levels or an integrative averaged daily cortisol level (24-h urine collection). However, none of these provide information about the long-term course of cortisol. In addition, diurnal variations in cortisol production and secretion may be a limiting factor in clinical interpretation (2).

In recent years, there has also been an increase in the investigation of cortisol levels in the context of mental disorders, as shown in a meta-analysis from 2018 (3). The authors demonstrated that the HPA axis appears to be altered in the context of mental disorders and that it may be helpful to consider biomarkers as an adjunct to conventional psychometric measures in psychological intervention studies (3). Biomarkers could be used as a marker of therapeutic progress and disease regression. Cortisol seems to be particularly suitable as a biomarker for mental processes.

As described in another systematic review on the influence of hormones on psychotherapies, it seems plausible that cortisol can influence psycho-affective and emotional functions through physiologically expressed glucocorticoid receptors in the central nervous system. The review attributes some importance to cortisol in the context of psychotherapy (4). Furthermore, it has been shown that cortisol may act as a moderating factor on psychotherapeutic processes (5). However, the extent of these influences require further investigation. The authors claim that the integration of repeated, stress-free hormone measurements, which have little influence on the therapy process, will allow conclusions to be drawn in the future about for whom and why psychotherapy works. They suggest that hair cortisol is a particularly suitable medium for this purpose (4).

The determination of cortisol in scalp hair seems to be a reliable parameter of the HPA axis (2, 6). In contrast to previous methods of cortisol determination, it allows the evaluation of the HPA axis activity over a longer period and represents long-term or continuous hormonal changes. Hair cortisol Concentration (HCC) sampling is non-invasive for the subject and therefore less susceptible to situational confounding, such as an increase in stress level due to the blood sampling (2).

With an average hair growth rate of approximately 1 cm per month, the most proximal centimeter represents the previous month’s HCC. Hair samples should be taken from the posterior vertex as close to the scalp as possible (6, 7). Two techniques are most commonly used to quantify hair cortisol: Liquid chromatography–tandem mass spectrometry (LC–MS/MS), a method for identifying and quantifying chemical compounds based on their fragmentation patterns and molecular masses, and Enzyme Immunoassays (EIA), which allows the detection of small amounts of antigens in a fluid sample based on the binding of the antigen to its specific antibody (6). No accepted standards have yet been established for hair cortisol norms, although several studies have attempted to generate reference data for different age groups as determined by LC–MS/MS or immunoassays (8–10).

As a helpful framework, biomarkers have been classified into diagnostic, prognostic, and intervention-related functions (11). The studies included in this review, used HCC as an intervention-related biomarker to map the effects of an intervention and predict post-intervention symptomatology.

### Background

The importance of HCC as a biomarker has been widely documented in various fields such as the measurement of stress or in the context of somatic diseases. The relationship between prolonged stress and HCC was demonstrated in a meta-analysis of chronic stress situations (12). In stress-exposed groups, the mean HCC increased by 22%. A positive correlation between HCC and stress is now widely accepted.

In addition, the measurement of the HCC may be relevant in several somatic diseases. One study showed that mean HCC were significantly increased in patients with Cushing’s syndrome (CS) (13). CS influences HCC, and HCC decreases during effective CS treatment. Increased HCC in patients with cyclic CS overlapped with the symptomatic phases of the disease. A previous study showed consistent results (14).

Studies on the relationship between HCC and overweight or obesity found significantly increased HCC levels in children with obesity (15). Furthermore, there is an increased risk of cardiovascular diseases with elevated HCC (16).

Regarding the association between maternal and infant HCC, several studies have provided interesting and sometimes conflicting results. Studies have shown a positive association between perceived stress and HCC in pregnant women, increased HCC in assisted reproductive technology, and increased HCC in the first trimester when a girl is carried to term (17–19). Regarding the mother–child relationship, one study found increased HCC in mothers with prolonged stress during pregnancy, but decreased HCC in their newborns (20). This interaction was confirmed in another study for the first trimester of pregnancy (21). Other studies showed highly interesting results on hair cortisol in pregnant women, but no clear interaction with fetal hair cortisol could be traced (22, 23).

Given the hypothesized critical role of prenatal stress in the early mother–child relationship, mediated by stress responses and subsequent neuroendocrinological changes, in contributing to the etiology of mental disorders in general, these findings suggest further investigation of the association between HCC and psychiatric or psychosomatic disorders (24). Previous studies suggest that HCC also plays an important role in mental disorders and underlying etiological relevant processes. For example, one study describes a dysregulation of the HPA axis in children with emotional and behavioral symptoms, which can be reflected by changes in HCC levels. Thus, children with emotional symptoms had significantly lower hair cortisol levels, whereas HCC levels were highest in older children with behavioral symptoms (25). Another study demonstrated that changes in PTSD symptomatology following trauma exposure could be predicted by HCC. A lower baseline HCC was associated with an increase in symptoms following a new trauma (26). A study of PTSD patients (based on a 12-months PTSD diagnosis), trauma patients (based on A1 and A2 criteria of a traumatic event), and healthy controls revealed significantly lower hair cortisol levels in the trauma and PTSD groups (27). In contrast, one study showed elevated HCC in PTSD patients when measured immediately after trauma exposure (28). Studies of the association between depressive disorders, which are highly prevalent in Western societies, and HCC have yielded conflicting results. Elevated hair cortisol levels were associated with persistent depressive symptoms (29). Significantly lower HCC levels have also been reported in adults with major depressive disorder compared to healthy controls (30). No differences in HCC were found between healthy individuals, patients with atypical depression, and patients with nonatypical depression (31). One of the few studies on depression and HCC in children showed that adolescents had higher HCC together with severe depressive symptoms, but only when they had high academic scores (32). This divergent picture of results is also evident in two meta-analyses: one of them concluded that depressive patients tend to have higher HCC than controls whereas PTSD patients show a trend toward lower HCC (33). The other one summarized that in most studies no differences in HCC were found between patients with major depression and healthy controls (34).

Thus, there is now a consensus in research community that environmental and mental stressors, as well as psychological distress, may ultimately be reflected in variations in HCC. The measurement of hair cortisol therefore seems to be a promising method to map chronic cortisol fluctuations that can occur in the context of psychiatric and psychosomatic diseases and thus also their treatment (35).

### Research questions

Based on the possible association between mental disorders and HCC, it is reasonable to assume that HCC also plays a role in the evaluation of psychological and neuropsychiatric interventions. However, this is still an evolving area of research, which explains the limited nature of the existing data. To date, there has been no review of the literature that classifies the importance of hair cortisol in the context of psychological or neuropsychiatric interventions. This literature review intends to give a first overview of the current state of research on the role of HCC in the context of psychological and neuropsychiatric interventions.

On the one hand, this review aims to explore the extent to which HCC is used as a trajectory parameter in research investigating the effects of psychological interventions on people with mental health problems. On the other hand, it is intended to answer the question whether the existing literature provides evidence of the extent to which hair cortisol concentration appears to be suitable as an outcome parameter for psychological and neuropsychiatric interventions by summarizing the existing literature in a structured way. The following research questions, which are formulated in a more general way according to the still preliminary character of the existing literature, shall be answered by the present work:

1. To what extent is HCC used as a predictive or trajectory parameter in psychological and neuropsychiatric intervention studies?
2. How do psychological and neuropsychiatric interventions affect HCC?

## Methods

This paper is a literature review based on the PRISMA statement (36). Accordingly, the literature research was developed through a qualitative approach and was guided by the PRISMA guidelines (36). A research protocol was developed, based on PRISMA suggestions (36). The review was not registered. By applying this methodology, it was possible to effectively limit the search for literature according to the objectives established. The following steps were employed: (1) definition of the research question, (2) setting up of the search strategy, (3) definition of the inclusion and exclusion criteria, (4) study selection, (5) data analysis.

### Search strategy

To address the research questions, the authors systematically searched the databases *Web of science, Google Scholar, PsycINFO* and *ResearchGate* for relevant literature from May to July 2022. The primary database utilized in this study was the *Web of Science Core Collection*. The other databases were employed to fulfill additional search queries, such as retrieving full texts or conducting targeted searches for specific articles. No date limit will be imposed on the search. Studies in languages other than English and German could not be included due to resource limits.

According to the research question, we identified appropriate keywords and linked them by using the Boolean operators “AND” and “OR.” The following combinations were used: (1) *hair cortisol* AND *review* or *meta-analysis*, (2) *hair cortisol* or *hair cortisol concentration* or *HCC* AND *psychotherapy* or *therapy* or *counseling* or *psychological therapy* or *psychosomatic treatment* AND *response* or *outcome* or *predictor* or *process* AND *PTSD* or *posttraumatic stress disorder* or *depressive disorder* or *depression* or *childhood maltreatment* or *anxiety disorder* or *somatic symptom disorder* or *psychosomatic disorder* or *major depression* or *fear* or *borderline disorder* or *personality disorder*.

Through these selected keywords, we aim to cover as broad a field of mental disorders as possible. However, the studies found in this way mainly examined HCC in the context of psychological and neuropsychiatric interventions in the presence of affective disorders. No relevant literature on hair cortisol and psychological or neuropsychiatric interventions in the context of borderline or other personality disorders could be found.

In addition, the reference section of the studies found by automatic search in the databases were manually searched for further relevant publications.

The authors also made a cursory search in gray literature. However, no suitable studies were found.

### Inclusion and exclusion criteria

Articles that considered the topic of hair cortisol and psychological or neuropsychiatric interventions in the title or abstract were included. The further criteria for studies to be included will be outlined below.

#### Study designs

Randomized controlled trials (RCTs), cluster RCTs, controlled clinical trials, controlled before-after studies, prospective, retrospective, and case–control studies as well as other forms of clinical trials were included. Time series analysis and different forms of qualitative research were also initially considered. The authors did not include non-empirical research studies, unpublished master-level dissertations, unpublished conference presentations and articles where no full text is available.

#### Participants

Studies examining humans of all ages; children and adolescents as well as adults were included. Animal studies were excluded.

#### Interventions

Different types of psychological interventions were considered for the present work, including widely-applied traditional forms of psychotherapy such as cognitive behavioral therapy (CBT), other forms of behavioral therapy, analytic and psychodynamic therapy, family therapy, or trauma focused therapy, but also interventions from the broader psychotherapeutic or neuropsychiatric spectrum such as stress-reducing therapies, mindfulness-based interventions, and electroconvulsive therapy (ECT). Studies that did not report any form of intervention were excluded.

#### Comparators

Various comparisons can be of interest for the present study and will be considered in the literature review. Studies that only measured before and after values of HCC are included as well as studies with an untreated control group or other forms of interventions as a control condition.

#### Outcome

All the included studies need to determine HCC as an outcome parameter. Both ways of quantifying HCC, LC-MS/MS and EIA, will be considered. Studies that did not quantify HCC were excluded.

### Selection of the studies

**Figure 1:**
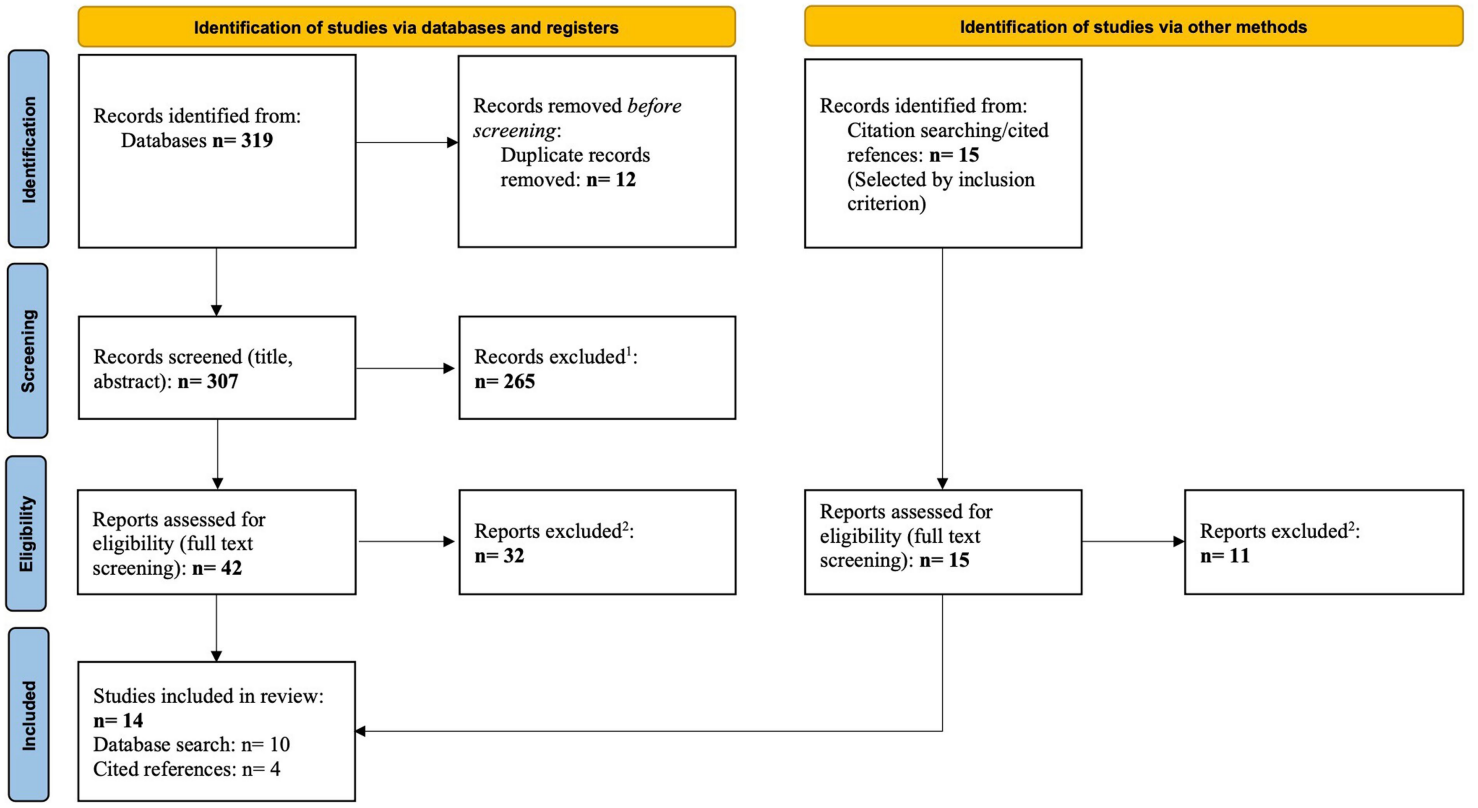
Flow diagram of study selection process [adapted according to PRISMA statement (36)].

According to the PRISMA guidelines (36) a flow chart was created (Figure 1), to illustrate the selection process of the relevant studies. The *Identification* phase shows the total number of articles found by database- and snowball-research, according to the keywords used. Before screening, the duplicates were removed. In the *Screening* phase, we applied the inclusion criteria to titles and abstracts. The exclusion criteria were then applied during the *Eligibility* phase. The criteria used in the corresponding phase are listed in the flow diagram. Finally, these steps resulted in 14 articles included in this review. The study selection process was conducted by two of the authors (VH, TB) independently.

### Quality assessment and data analysis

Based on the selected studies, we read each article with particular attention to describe the significance of HCC and the potential predictive value of HCC in psychological and neuropsychiatric interventions. Microsoft Excel was used to manage and organize the relevant extracted information from the studies. Subsequently, these were then transferred into a well-structured table containing the most important data and results of the publications considered. If specified in the publications, the individual hair cortisol values measured are also listed. The table is divided into a part with the studies that mainly focus on the longitudinal changes of HCC in the course of a psychological or neuropsychiatric intervention and a second part with the studies on the predictive power of HCC for the treatment outcome (see Tables 1A,B). The studies and their results are moreover presented, summarized, and evaluated in detail in the following.

**Table 1:** Overview of reviewed articles **(A)**—concerning HCC as a longitudinal parameter and **(B)—**concerning HCC as a predictive parameter.

Authors, country	Title	Sample	Treatment and measures	HCC and method of HC-measuring	HCC results
**Overview of reviewed articles (A)—concerning HCC as a longitudinal parameter**					
Romero-Gonzalez et al. (37), Spain	Effects of cognitive-behavioral therapy for stress management on stress and hair cortisol levels in pregnant women: A randomized controlled trial	*n* = 78 pregnant women	CBT: 8 weeks, 1.5–2 h per week in groups of 4–5 patients	HCC (no specific unit given; presumably in pg./mg):	Reductions in HCC, specific pregnancy stress levels and perceived stress in CBTg-group
		Median age = 33.07 years (SD = 4.63)		CG: T_0_ = 5.50 (0.91), T_1_ = 5.62 (0.86)	
RCT (single blind)			PSS; PDQ; SCL-90-R; CD-RISC	CBTg: T_0_ = 4.78 (0.97), T_1_ = 4.47 (0.94)	
		CG: *n* = 39 of 78 (no CBTg)		Salivary ELISA cortisol kit	
				Sample collection pre- and post-intervention	
Puhlmann et al. (38), Germany	Contemplative Mental Training Reduces Hair Glucocorticoid Levels in a Randomized Clinical Trial	*n* = 322 (197 women)	Mental training	Mean HCC in pg/mg (SD)	HC levels remained stable in RCC
		Median age = 40.74 years, SD = 9.24, range 20–55 years		T_0_ = 7.46 (8.97) T_1_ = 5.81 (6.85) T_2_ = 4.59 (5.18) T_3_ = 4.66 (3.51)	With mental training HCC decreased until 6 months into the training regimen
RCT (Open-label efficacy trial)		CG: allocation to either one of two 9-month training cohorts that completed all 3 training moduls (TC1 *n* = 48, TC2 *n* = 62), a 3-month Affect-only training cohort (TC3 *n* = 49) or a retest control cohort (RCC *n* = 68)	PSS; global stress score of the TICS	Liquid chromatography-tandem-mass spectrometry	final 9-month measurement, HCC stabilized at this lowered level
				Count of hair samples: T_0_ = 217, T_1_ = 157, T_2_ = 136, T_3_ = 150	Suggesting that greater training compliance led to stronger HC reduction
Poehlmann-Tynan et al. (39), United States	Cognitively Based Compassion Training for parents reduces cortisol in infants and young children	*n* = 38 parents and their children (33 mothers; 19 boys, 19 girls)	CBCT: total of 20 h	No hair cortisol value given	Postinterventional parent HCC: no intervention effect
RCT		median age: children = 3.2 years (9 month–5 years 4 month), parents = 36.7 years		LC–MS/MS	Postinterventional child HCC: children of CBCT intervention parents less HCC at postintervention
		CG: *n* = 14	C-SOSI; PHD; PSI-SF; SCS; FFMQ	pre- and post-intervention	Average child HCC in the intervention group decreased, whereas in the control group increased
Woud et al. (40), Germany	The Effects of Modifying Dysfunctional Appraisals in Posttraumatic Stress Disorder Using a Form of Cognitive Bias Modification: Results of a Randomized Controlled Trial in an Inpatient Setting	*n* = 80 PTSD patients	CBM-APP: 8 Weeks	HCC CBM-APP-group (log_10_ pg./mg), Median:	No effect of CMB-APP training on HCC at any time
		Median age = 42.41 years, 36 women (CBM-Group)		PT = 0.59 PD = 0.59 FU = 0.59	
				HCC CG (log_10_ pg./mg), Median:	
RCT		CG = PVT (Peripheral Vision Task) = sham training: *n* = 40	PTCI, PCL-5, CEQ	PT = 0.31 PD = 0.34 FU = 0.43	
		Median age = 39.05 years, 34 women		LC-MS/MS	
				3 times: pretraining (PT), predischarge (PD), 3-months follow-up (FU)	
Iglesias et al. (41), Argentina	Hair cortisol: A new tool for evaluating stress in programs of stress management	n_1_ = 83; completed the full program: n_2_ = 37 (31 women, 6 men)	90–120 min stress management interventions for 10 weeks	Mean HCC in pg/mg:	HCC from the last session was significantly lower than HCC from the first session
		median age_1_ = 35.7+/− 12.2 years		Pre = 226.3	
Quantitative non-randomized study		median age_2_ = 34.0 +/− 10.06 years	STAI	Post = 113.0	
		CG: /		Immunoassay (Roche Cobas e-411 Cortisol assay)	
Dajani et al. (42), Jordan	Hair cortisol concentrations in war-affected adolescents: A prospective intervention trail	*n* = 733 (411 syrian refugees, 322 Jordanians)	Intervention based on profound stress attunement	Mean baseline HCC (no specific unit given)	At baseline: no relationship between HCC and trauma exposure
				PTSD = 0.86	PTSD-Patients had higher baseline HCC
RCT		Age = 12–18 years	Trauma Event Checklist; CYRM; PSS; CRIES	No PTSD = 0.79	
		Sample split into those who had experienced < 4 (CG) and > 4 average trauma (intervention group)		ELISA	intervention led to a normalization of HCC
Kische et al. (43), Germany	Applied relaxation and cortisol secretion: findings from a randomized controlled indicated prevention trial in adults with stress, anxiety, or depressive symptoms	*n* = 277	AR group training: 10 sessions of 60–90 min	Mean HCC in pg/mg: Intervention:	No effect in IG on HC change
				Pre = 5.83	
		HCC-Sample: *n* = 162 [Intervention group (IG) = 76]		follow-up = 6.87	
RCT		Median age IG = 36 years	DASS-21	CG: pre = 4.43	No significant association between frequency of AR use and cortisol change in IG
		CG: n_HCC_ = 86		follow-up = 5.38	
		Median age = 33.7 years		Immunoassay, sampling at pre-assessment and at follow-up	
Mickey et al. (44), United States	Cortisol trajectory, melancholia, and response to electroconvulsive therapy	*n* = 39 patients with treatment-resistant depression	ECT: mean number of treatments: 10.5	No hair cortisol value given	ECT response is associated with long-term alterations in basal cortisol levels
Observational study		Median age = 49.4 years (SD = 17.0), range 19–80 years	MINI, HDRS, CGI	Immunoassay	Responder showed an increase of HCC, non-responder a decrease
		CG: responder vs. non-responder			
Koncz et al. (45), Hungary	Mediation interventions efficiently reduce cortisol levels of at-risk samples: a meta-analysis	*n* = 34 studies; only 2 studies concerning HCC, together 223 participants			The effect size of the two studies showed no effect of mindfulness-based and focused attention (FA) interventions (used in both studies) on HCC
Meta-analysis					

**Overview of reviewed articles (B)—concerning HCC as a predictive parameter**					
Fischer et al. (46), United Kingdom	Hair cortisol and childhood trauma predict psychological therapy response in depression and anxiety disorders	n = 89 (83 women) median age = 34 years	Treatment: all therapeutic approaches for depression & Anxiety recommended by NICE guidelines (incl. CBT)	Median HCC = 153 (321.2) pg./mg	Non-Responder in terms of depression and anxiety: lower pre-treatment HCC (131.9 (214.1) pg./mg vs. 153 (409) pg./mg; 131.9 (214.1) pg./mg vs. 150.6 (245) pg./mg)
Cohort study		“CG”: groups of different severity of symptoms	MINI; CTQ; PHQ-9; GAD-7	Immulite Immunoassay analyzer	No differences in HCC when comparing patients without vs. with childhood abuse and neglect
Steudte-Schmiedgen et al. (47), Germany	Hydrocortisone as an adjunct to brief cognitive-behavioral therapy for specific fear: Endocrine and cognitive biomarkers as predictors of symptom improvement	*n* = 36 spider-fearful individuals; HCC obtained from 30 participants; sample split in 1 placebo and 1 hydrocortisone group	Single-session exposure-based CBT (45 min)	Baseline HCC (M, SD; no specific unit given)	Baseline HCC no predictor of change on/in SAS, FSQ, BAT speed and distance or threat bias
				Presumably in pg./mg:	
				Placebo = 7.68 (4.56)	
				Hydrocortisone = 7.83 (8.00)	
RCT (double-blind)		Median age = 24.25 years (placebo); 25.12 years (hydrocortisone)	BDI-II; PSS; STAI; SAS; FSQ, BAT	LC-MS/MS	Baseline HCC not predictive of treatment response and did not regulate the efficacy of hydrocortisone treatment
				Sample collection on intervention day	
Basso et al. (48), Germany	Psychological Treatment Effects Unrelated to Hair-Cortisol and Hair-BDNF Levels in Chronic Tinnitus	*n* = 94 patients with chronic tinnitus (53 women)	Compact Multimodal Tinnitus-Specific CBT (on average over 4.78 days)	No hair cortisol value given	Therapy reduced tinnitus-related distress and perceived stress levels; HCC did not reflect the improvements
		mean age = 50.96 years			
Longitudinal study		HCC-sample *n* = 91	TQ, PSQ-20, HADS, SOMS, STAI, PDS, SF-12	ELISA, sampling at pretreatment and at 3-months follow up	Baseline HCC was not associated with psychological treatment outcomes and had no predictive value
		CG: /			
Hummel et al. (49), Germany	The predictive role of hair cortisol concentrations for treatment outcome in PTSD inpatients	*n* = 52 female PTSD inpatients	Multimodal trauma-focused psychotherapy for PTSD	Mean HCC in pg/mg (SD):	Overall HCC increase from Pre to FU,
				Pre = 6.36 (6.75)	Pre-treatment HCC not associated with changes in PDS scores or from Pre to FU
		Median age = 42.60 years (10.54)		Post = 5.76 (3.84)	lower pre-treatment HCC associated with reduced symptom reductions; no predictive associations for changes in SCL-90-R from Pre to FU
Prospective study		CG: /	PDS; SCL-90-R; GSI; BDI; CTQ; FDS-20	FU = 8.64 (7.08)	No predictive value of HCC for PTSD symptom changes
				Liquid chromatography–tandem mass spectrometry; 3 times: treatment entry (Pre, *n* = 52), discharge (Post, *n* = 42), follow-up (FU, *n* = 27; on average 5 month later, mean = 140 days)	Changes in HCC were not predictive for changes in any symptom measured
Baeten et al. (50), Belgium	Hair cortisol in patients with a depressive episode treated with electroconvulsive therapy	*n* = 62 (51 women)	ECT: 2× a week for 3 months	Median HCC in pg/mg = 4.4 (IQR 4.5)	HCC were not significantly higher in ECT responder than in non-responder
Single site prospective study		Median age = 60 years (SD = 14)			
		CG: subgroups depending on severity of symptoms	PDAS; CORE Assessment of Psychomotor Functioning; HDRS, BFCRS; MINI	LC-MS/MS	High HCC was not associated with a beneficial ECT outcome

BAT, Behavioral Approach Test; BDI/BDI-II, Beck Depression Inventory; BFCRS, Bush-Francis Catatonia Rating Scale; CD-RISC, Connor Davidson Resilience Scale; CEQ, Credibility/Expectancy Questionnaire; CEVQ, Childhood Experiences of Violence Questionnaire; CG, control group; CGI, Clinical Global Impression improvement scale; CRIES, Children’s Revised Impact of Event Scale; C-SOSI, Calgary Symptoms of Stress Inventory; CTQ, Childhood Trauma Questionnaire; CYRM, Child and Youth Resilience Measure; DASS-21, Depression-anxiety-stress-scale; ECT, electroconvulsive therapy; FDS-20, adaption of Dissociative Experience Scale; FFMQ, Five Facet Mindfulness Questionnaire; FSQ, Fear of spider Questionnaire; GAD-7, Generalized Anxiety Disorder Scale; GSI, Global Severity Index; HADS, Hospital Anxiety and Depression Scale; HCC, hair cortisol concentration; HC, hair cortisol; HDRS, Hamilton Depression Rating Scale; HE, hair cortisone; MINI, Mini-International Neuropsychiatric Interview; PCL-5, PTSD checklist for DSM-5; PDS, Posttraumatic Diagnostic Scale; PDAS, Psychotic Depression Assessment scale; PDQ, Pregnancy Distress Questionnaire; PDS, Posttraumatic Diagnostic Scale; PHD, Parenting Daily Hassles Scale; PHQ-9, Patient Health Questionnaire; PSI-SF, Parenting Stress Index-Short Form; PSQ-20, Perceived Stress Questionnaire; PSS, Perceived Stress Scale; PTCI, Posttraumatic Cognitions; RCT, randomized control trial; SAS, Spider Anxiety Screening; SCL-90-R, Symptom Checklist-90-Revised; SCS, Self-Compassion Scale; SF-12, Short Form-12 Health Survey; SOMS, Screening of Somatoform Disorders; STAI, State–Trait Anxiety Inventory; TQ, Tinnitus Questionnaire.

To evaluate the risk of bias and the quality of the studies included, we used the Mixed methods appraisal tool [MMAT; (51)]. The MMAT is specifically designed for systematic mixed studies reviews and was therefore chosen for the present research. As the MMAT appraisal tool [see Table 2; (51)] shows, the methodological quality of the studies, although predominantly high, is heterogeneous. The included studies consist of 13 quantitative trials and one meta-analysis (not assessed by MMAT). Seven of the studies could be identified as randomized controlled trials. The remaining six are different forms of quantitative non-randomized studies such as cohort studies, observational or prospective trials. For all non-randomized studies and for four of the randomized studies, at least four of five questions of the MMAT quality assessment could be answered with “yes.” However, some limitations were observed or described by the respective authors themselves. Among them are a small sample size, determination of HCC at only one or two time points or a short follow-up period. Considering the mostly small number of subjects, it is nevertheless positive to note that the samples of most studies were heterogeneous and thus represented a good cross-section of the underlying population. In the presence of a control group, the subject groups were comparable at baseline in all these studies. Only in three of the non-randomized trials, the study population consisted predominantly or entirely of women. In one, it is explicitly stated that the selection of the participants may not be representative. Therefore, for these studies, the question of the MMAT tool as to whether the sample was representative, had to be answered with “no.”

**Table 2:** Quality appraisal of the studies using the mixed methods appraisal tool (MMAT).

MMAT category of study designs	Study	Response					MMAT methodological quality criteria [Hong et al. (37)]
1. Screening questions	For all studies	S1.	Y				S1. Are there clear research questions?
		S2.	Y				S2. Do the collected data allow to address the research questions?
2. Quantitative randomized controlled trials		2.1	2.2	2.3	2.4	2.5	
	Romero-Gonzalez et al. (37)	Y	Y	Y	N	Y	2.1. Is randomization appropriately performed?
	Steudte-Schmiedgen et al. (47)	Y	Y	Y	Y	Y	2.2. Are the groups comparable at baseline?
	Puhlmann et al. (38)	C	Y	Y	C	Y	2.3. Are there complete outcome data?
	Poehlmann-Tynan et al. (39)	C	Y	Y	C	Y	2.4. Are outcome assessors blinded to the intervention provided?
	Woud et al. (40)	Y	Y	Y	Y	Y	2.5. Did the participants adhere to the assigned intervention?
	Dajani et al. (42)	C	Y	Y	C	Y	
	Kische et al. (43)	Y	Y	Y	N	Y	
3. Quantitative non-randomized		3.1	3.2.	3.3	3.4	3.5	
	Fischer et al. (46)	N^1^	Y	Y	Y	Y	3.1. Are the participants representative of the target population?
	Basso et al. (48)	Y	Y	Y	Y^2^	Y	3.2. Are measurements appropriate regarding both the outcome and intervention (or exposure)?
	Hummel et al. (49)	N^1^	Y	Y	Y	Y	3.3. Are there complete outcome data?
	Iglesias et al. (41)	N^1^	Y	Y	Y	Y	3.4. Are the confounders accounted for in the design and analysis?
	Mickey et al. (44)	N	Y	Y	Y	Y	3.5. During the study period, is the intervention administered (or exposure occurred) as intended?
	Baeten et al. (50)	Y	Y	Y	Y	Y	

Y, Yes; N, No; C, Cannot tell. ^1^study population consist predominantly or entirely of women; ^2^Although many confounding factors, such as socio-demographic or lifestyle factors, were taken into account, it was not possible to consider all of them (e.g., not considered: comorbidities, medication).

The non-randomized studies in general must be assessed with a high risk of bias due to the lack of randomization and the absence of a control group in some of them.

In the case of three of the randomized controlled trials, it should be noted negatively that the randomization process was not adequately described. Furthermore, it is not clear whether blinding took place. Another randomized controlled trial did not use blinding of the researchers and participants at all, but this circumstance was clearly described. However, this leads to a high risk of detection bias.

Due to the still small number of studies considered and the heterogeneity of their methodological quality, we decided not to conduct a meta-analysis.

## Results

As shown in Figure 1, the initial, automatic search of the above-mentioned databases produced 319 results. First, 12 duplicates were filtered out using the *Find Duplicates* function in the literature management program EndNote X9. The remaining 307 articles were then screened by title and abstract. In this process, 265 were found to be ineligible for this review. In a further step, the full texts of the remaining 42 studies were screened and evaluated. This resulted in the exclusion of 32 publications.

In addition, as mentioned above, a hand-search was conducted in the reference section of the previously selected studies. Initially, 15 studies were found to be usable. In the full-text screening, 11 of these results had to be excluded. The exclusion criteria can be found in the methods section and in Figure 1. To illustrate the exact selection process, a flowchart was created as described in the methods section (see Figure 1).

The selection process thus resulted in a total number of 14 studies, which are considered and described below.

### Overview of included studies

The 14 studies reviewed are summarized in Tables 1A,B. Regarding the research questions, some of the studies address the effects of well-established psychotherapy treatments such as cognitive behavioral therapy (CBT), while others examine the effect of psychological interventions in a broader sense.

The oldest study considered here was published in 2015, the most recent ones are from 2022. The vast majority of the included publications come from Europe (Germany, United Kingdom, Spain, Belgium, Hungary). Two were from the USA and one each from Argentina and Jordan.

These 14 studies collectively involved a total of 1,916 participants. The number of participants varied significantly and ranged from 30 to 733. The average age of the study population also varied widely, ranging from adolescents to an average age of 60 years. Mostly, however, it was in the range of 30 to 45 years. Accordingly, studies that included only adolescents/children, only adults, or both age groups as participants were included. Only one study included females only. The other study populations were of mixed gender.

The 14 studies encompassed in this review followed diverse methodological approaches. One is a meta-analysis, and the others are quantitative trials of various types.

Consistent with the defined research questions, the authors included nine studies focusing on longitudinal changes in HCC over the course of a psychological or neuropsychiatric intervention and five studies concentrating on the predictive power of hair cortisol on treatment response.

### Results on longitudinal changes of hair cortisol

Regarding longitudinal changes in HCC over the course of psychological or neuropsychiatric interventions, one study investigated the effects of CBT, a well-established form of psychotherapy, on HCC. This randomized controlled trial of 78 pregnant women, divided in an experimental and a control group, examined the efficacy of CBT for stress management in reducing psychological stress and HCC. In the therapy group, a reduction in HCC, specific pregnancy stress scores, and general stress levels (lower PDQ-, PSS-, SCL-90-R-, PSDI-scores) was found. According to the authors, the decrease in these psychological and endocrinological stress parameters reflects the effectiveness of CBT in pregnancy (37).

With regard to less intensive psychological interventions, the effects of different mental training programs on long-term endocrine and psychological stress parameters were investigated. Regardless of the training content, HCC values decreased continuously in 159 participants up to 6 months post-treatment and were significantly reduced compared to the 68 controls. After 9 months, HCC stabilized at a lower level or slightly increased compared with the baseline (38). Similarly, an effect of Cognitively Based Compassion Training (CBCT) on HCC was demonstrated. Therefore, 25 parents undergo an 8-week CBCT program and parental *and* child HCC was subsequently determined. Interestingly, there was no significant interventional effect on parental HCC. However, the children had lower HCC after their parents’ treatment compared with controls, where concentrations increased (39). Moreover, a Cognitive Bias Modification for Appraisals intervention failed to have any effect on HCC in the study population of 80 PTSD-patients (40).

Two of the three included studies, which focused primarily on the effect of stress-reducing interventions, found an association between treatment and HCC. A 10-week stress management intervention for 83 adults resulted in significantly lower HCC at the last, compared to the first appointment (41). In a group of 411 underage Syrian refugees and 322 Jordanian adolescents, some with PTSD, the influence of a psychosocial, stress-reducing intervention on cortisol production was analyzed. Regardless of gender, origin, or trauma exposure, this form of therapy resulted in an average reduction in HCC of one third across all groups. Looking at the groups individually, a decrease in HCC was evident in the group of adolescents with cortisol hypersecretion and medium secretion, and an increase was measured in adolescents with hyposecretion (42). No association was shown between HCC and an applied relaxation group training in a study with 277 adults with symptoms of stress, anxiety, or depression. Neither for the intervention in general nor for the frequency of therapy significant changes in HCC could be found (43).

Electroconvulsive therapy (ECT) has established itself as a form of neuropsychiatric treatment, especially for severe depression (52). In one study of the effects of ECT on HCC 39 patients with treatment-resistant depression were subjected to an average of 10.5 ECT sessions. In responders, HCC tended to increase, in non-responders it decreased (44).

Finally, a meta-analysis examined the effects of meditation on cortisol. However, only two studies quantified HCC. In both, there was no effect of mindfulness-based and focused attention interventions on HCC (45).

### Results on predictive power of hair cortisol on treatment response

Five studies on this topic could be found. Three of them used variants of cognitive behavioral therapy. One of them provided results indicating a *predictiv*e value of HCC. In 89 patients with depression and anxiety disorders, it was investigated whether HCC was a predictive parameter for treatment outcome. The treatment included all NICE guideline-recommended treatment approaches for depression and anxiety, including CBT. Non-responders had a lower HCC *before* the start of therapy (46). In a further study, 36 patients with arachnophobia participated in a single 45-min CBT session. The research group subsequently assessed the value of the basal endogenous glucocorticoid secretion regarding treatment outcome. However, basal HCC did not emerge as a significant predictor of changes in 1-month follow-up scores on the anxiety questionnaires (Self-Rating Anxiety Scale, Fear of Spiders Questionnaire) and therapy response (47). In addition, the effects of tinnitus-specific CBT on HCC and other parameters were examined in 91 patients with chronic tinnitus. HCC did not reflect the positive effects of the CBT on tinnitus-related distress and perceived stress levels. Thus, HCC was not related to with the outcome of this kind of therapy (48).

In terms of less established psychological interventions, a study examined HCC in the context of multimodal trauma-focused inpatient psychotherapy for PTSD patients. Hair samples from 52 female PTSD patients were analyzed at different examination times. HCC measured at admission and discharge did not differ. Lower HCC before therapy could *predict* less improvement in clinical symptoms, but this relationship was no longer statistically significant after adjustment for baseline dissociative symptoms. Thus, there was *no clear predictive* value of HCC with respect to changes in PTSD symptomatology (49).

In another study investigating the association between ECT and HCC, 62 patients with varying degrees of major depression were treated with ECT twice a week for 3 months. Here, HCC was not significantly elevated in the responder group. Increased hair cortisol levels therefore do not appear to be associated with better therapy outcome (50).

## Discussion

The aim of this review was to summarize the current state of research on the effects of different psychological and neuropsychiatric interventions on HCC and to investigate the extent to which hair cortisol can be altered in such types of interventions. Although only a few studies have been published to date, there has been a general trend toward increased relevance of HCC measures in the context of psychological and neuropsychiatric interventions.

HCC has been used to assess stress-related interventions such as relaxation training and, in fewer studies, for traditional forms of psychotherapeutic interventions such as CBT.

Looking at these publications, the reported study results are still rather inhomogeneous. As CBT is a well-established form of psychotherapy, the results of these studies are of particular interest. Two studies showed different associations between CBT and HCC. One study showed a decrease in HCC in the treatment group (37). In the second study, non-responder had lower baseline HCC *before* intervention. Thus, reduced HCC appears to be associated with a lower likelihood of treatment success (46). These studies are in contrast to other studies investigating HCC in CBT, which found no predictive value of HCC for treatment outcome and no direct relationship between HCC and therapy results (47, 48). Thus, how and if HCC is affected by this form of therapy cannot yet be adequately demonstrated. Further comparable and well-designed studies are needed to confirm the reported results.

Data on different relaxation methods, mental training, and ECT are also mixed. Half of the included studies found an association between the different interventions and a predictive potential in form of a longitudinal decrease in HCC.

In the context of a multimodal trauma-focused inpatient psychotherapy, there was no predictive value for HCC changes in terms of PTSD symptoms improvement (49). Various mental training interventions in one study and stress-reduction interventions in two studies led to sustained reductions in HCC (38, 41, 42).

The variability of HCC in ECT appears to be puzzling. Therapy responders showed increased HCC in one study (44). However, this result could not be confirmed, and a high HCC was not associated with a positive treatment outcome in another, more recent study (50).

In summary, there is a trend toward a *reduction* in HCC with psychological and neuropsychiatric interventions, as no study showed a clear *increase* in HCC after the intervention, but several found a reduction in cortisol levels. As stress, as reflected by the cortisol levels, is an important underlying factor in the development of mental disorders, a reduction of cortisol levels seems to be of particular importance in alleviating the symptoms of disorders such as depression or PTSD. This highlights the importance of establishing a standard biomarker to measure the impact of psychological interventions.

However, the results are still rather inconclusive. The explanation may be complex. One important aspect to consider seems to be that the effects of psychotherapy are not linear. Psychotherapy can often be upsetting and challenging within a given process. Thus, it may have an immediate stress-inducing effect, with the relieving effect coming later. The immediate effect of psychotherapy may also vary depending on the type of therapy and the invasiveness of the therapeutic interventions. The collection of catamnestic data may therefore be of particular importance in mapping the long-term effects of interventions. The lack of effects in some of the reported studies may be explained by the fact that they involved low-intensity stress management training and the like, which did not produce profound changes in the patients that resonated down to the biological level.

The most important reason for the heterogeneous data may be the methodology used in the included studies and their varying quality. Due to the quality criteria, only a small number of publications could be included, which underlines the novelty of the research field. Hair cortisol as a biomarker for psychological and neuropsychiatric interventions should be considered as little established in a still evolving research field, which required the inclusion of studies of heterogeneous quality.

Some methodological limitations of HCC measurement must be kept in mind, which limit the validity and significance of the study results presented above. The comparability of all included studies is limited due to differences in age structure, numbers of participants and type of intervention. In addition, the reported divergent results may be due to different study designs. Additionally, gender differences in HCC, with higher values in adult men, may influence the discrepancy (8). Age *per se*, and hormonal changes, such as pregnancy, influence HCC. Higher hair cortisol levels have been found in young children and older adults (53). Thus, when evaluating the effect of therapy on hair cortisol or when assessing the predictive power of HCC, the age of the participants is an important potential confounding factor.

Furthermore, the different measurement methods of HCC limit the comparability of the reviewed studies. It could be shown that the two methods of measurement were highly correlated with each other, but that immunoassays showed a constant tendency toward higher values (54). In addition, there is inter-laboratory variation in HCC detection (12), which explains why it has not been possible to establish generally accepted reference values. Nevertheless, the establishment of normative HCC data appears to be of critical importance as long as the reported variance in the treatment-related outcomes may be due to different methodological approaches to HCC determination. Normative values could thus contribute to a better comparability of the results.

Nevertheless, a possible effect of various psychological interventions on HCC as well as some predictive value of hair cortisol in the context of such treatment modalities cannot be excluded. However, these results are still inconclusive and need to be investigated and replicated in further studies.

## Conclusion

In summary, HCC may be a valuable parameter for monitoring psychological and neuropsychiatric interventions in psychotherapy research. HCC seems to be particularly suitable for this purpose because it consists of aggregated cortisol levels over a defined period of time and represents a stress-related aspect of any psychological intervention or neuropsychiatric treatment. At present, the extent to which HCC can be used for these purposes cannot be clearly determined, not least because of the inconsistent data situation. Thus, the current state of research reflects only a fundamental orientation. However, it indicates that the documentation of changes in HCC during the therapeutic process seems promising and most authors consider it useful to include psychoendocrine biomarkers in psychological and neuropsychiatric intervention studies. Nevertheless, the potential of HCC for true psychological or neuropsychiatric investigations needs to be solidified by further studies with greater consideration of the parameters and study design requirements mentioned above. In the long run, this will hopefully define a clearer direction on the impact of psychological interventions on HCC and thus contribute to the relevance of hair cortisol as a standard psychobiological parameter. Psychopharmacological studies and larger epidemiological surveys of stress in a general population would benefit as well.

The psychoendocrine approach to psychotherapy and psychological interventions may help to compare the effects of different psychotherapeutic methods and to establish differential indications for psychosomatic/psychotherapeutic treatment modalities. Especially for the psychosomatic field, the combination of *psychological* and *somatic* parameters to measure the therapeutic effect seems intuitive and highly relevant.

## Data availability statement

The original contributions presented in the study are included in the article/supplementary material, further inquiries can be directed to the corresponding author.

## Author contributions

TB, BB, and EP contributed to conception and design of the study. VH contributed to literature research and wrote the first draft of the manuscript. All authors contributed to the article and approved the submitted version.
